# Surgical management of cervical kyphosis in larsen syndrome. Case report and review of literature

**DOI:** 10.1016/j.amsu.2022.103372

**Published:** 2022-02-11

**Authors:** Mohammed Armouti, Hawazen Hirbawi, Mutaz Jadaan, Hasan Hashem, Baha'eddin A. Muhsen

**Affiliations:** a Department of Orthopedic Surgery, Abdali Hospital, Amman, Jordan; b Department of Surgery, Istishari Hospital, Amman, 11184, Jordan; c Department of Pediatrics, King Hussein Cancer Center, Amman, 11941, Jordan; d Department of Neurosurgery, King Hussein Cancer Center, Amman, 11941, Jordan

**Keywords:** Larsen syndrome, Cervical kyphosis, Kyphoscoliosis, Corpectomy, Spinal stabilization

## Abstract

**Introduction:**

and importance: Larsen syndrome is a rare genetic disorder that is characterized by multiple joint dislocations, flat faces that can also be referred to as “dish face”, kyphoscoliosis, and anomalies of the vertebrae. Patients with this syndrome frequently develop various spinal deformities, one of them being kyphosis of the cervical spine. This deformity can lead to serious health manifestations if not surgically treated.

**Case presentation:**

We report a case of a 6-month-old female, diagnosed with Larsen syndrome. She presented with progressive upper and lower limbs spasticity, flexed neck, and bilateral resistant developmental dysplasia of the hip. A C3 corpectomy with iliac crest allograft was done and 2.7 plates with screws in C2 and C4 were placed. An abduction brace (Pavlik harness) was used for 3 months after the surgery to prevent early collapse.

**Clinical discussion:**

Our patient was the youngest patient reported in the literature to be operated on. Although the type of surgery for patients with Larsen who suffer from spinal deformities is dictated by the severity of the deformity; the literature agrees that surgical intervention is the most important step in its management.

**Conclusion:**

If cervical kyphosis in a patient with Larsen syndrome is left untreated; the progression of the condition can eventually lead to paralysis. Early surgical correction can spare the patient future deterioration due to chronic cord compression.

## Highlights

• Patients with this syndrome frequently develop kyphosis of the cervical spine.
• Surgical intervention is the most important step in managing this condition.
• We report the youngest case of a patient with Larsen to be surgically operated on.
• Early surgical intervention results in a better prognosis and quality of life.

## 1 Introduction

Larsen syndrome was first described in 1950 by Loren J. Larsen [1]. He described the first series of cases with distinctive facial features, multiple joint dislocations, and spinal anomalies. Larsen syndrome is a rare genetic disorder characterized by the triad of odontoid hypoplasia, small bullet-shaped vertebral bodies, and typical flat “dish” faces. Patients with this syndrome frequently develop various spinal deformities, such as kyphotic deformity of the cervical spine and thoracolumbar scoliosis [2]. From the many syndromes associated with craniovertebral junction instability, Larsen syndrome is the most challenging one to treat [3].Cervical kyphosis is one of the deformities that come with Larsen syndrome and is potentially the most serious manifestation due to the risk of life-threatening paralysis [4]. Early surgical stabilization of Larsen syndrome plays an important role in the management of this syndrome [5]. This paper has been reported in line with the SCARE criteria [6].

## 2 Case presentation

We present the case of a 6-month-old female, diagnosed with Larsen syndrome. She presented with progressive upper and lower limbs spasticity, flexed neck, and bilateral resistant developmental dysplasia of the hip since birth. History and physical exam revealed delayed developmental milestones with spasticity in upper and lower limbs. A whole exome sequencing revealed a heterozygous variant in the FLNB gene consistent with autosomal dominant Larsen syndrome.

**Fig. 1:**
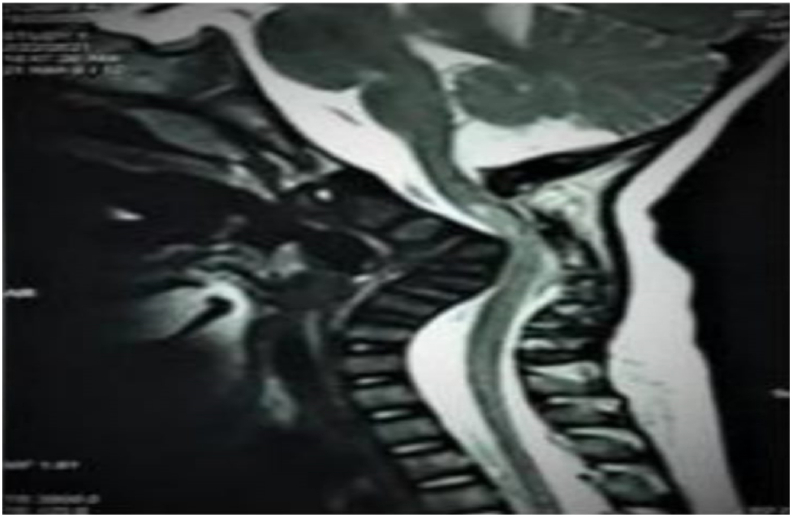
Cervical spine MRI, Sagittal T2 sequence, showing kyphotic deformity of the cervical spine causing significant cord compression and T2-hyperintense signal abnormality related to myelomalacic changes.

**Fig. 2:**
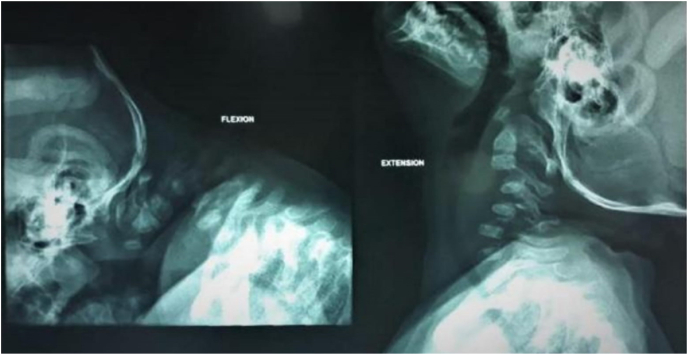
Dynamic Neck X-ray (Flexion and extension) showing cervical kyphosis, anterior wedging of C3, C4 associated with instability.

A brain MRI was done and it didn't show any abnormalities. However, the cervical spine MRI revealed a deformity of the cervical spine causing significant cord compression (Shown in Fig. 1). A dynamic neck x-ray was done and showed cervical kyphosis, and anterior wedging of C3 and C4 associated with instability (Shown in Fig. 2). An ultrasound was also done to confirm the diagnosis. Other musculoskeletal findings include hind feet, congenital metatarsus (Primus) varus, ulnar deviated wrists, generalized joint hyperlaxity and muscular hypertonia.

### 2.1 Surgery

**Fig. 3:**
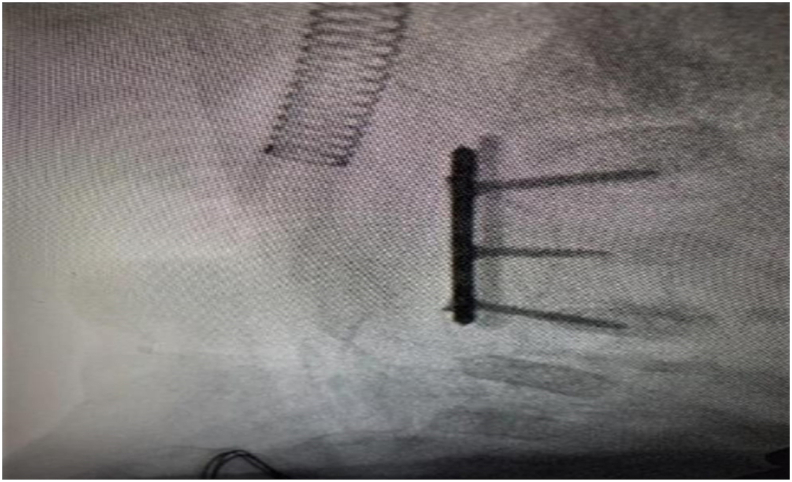
Intraoperative X-ray showing properly placed iliac bone graft after C3 corpectomy and fusion C2–C4 with plate and screws with correction of the kyphotic deformity.

Under general anesthesia and hyperextension of the neck. After the neck was prepped and draped, a right transverse incision at the level of C3 using lateral fluoroscopic image to identify level was performed. After subcutaneous tissue and platysma muscle dissection, the carotid artery sheath was identified and dissected to reach the cervical spine; where a small right angle was used to retract tissues including longus coil muscles. C3 was identified by lateral fluoroscopy as well as C2-3 and C3-4 disc space. The microscope was used to magnify the field and corpectomy was started using a size 1 high-speed drill and Kerrison punch. Then, the space between C2 and C3 was measured by a ruler. Iliac crest allograft was prepared according to the space measured. Three holes, titanium, and 1.5 mm locking plate were used. The middle hole was used to fix bone graft to the plate by 1.5 *8 mm screw. The iliac crest graft was fitted inside the space and a 1.5*10 mm screw was inserted in C2 and C4. After, irrigation, closure of platysma, subcutaneous and skin was performed. An Intraoperative X-ray was done showing properly placed iliac bone graft after C3 corpectomy and fusion C2–C4 with plate and screws and with correction of the kyphotic deformity (Shown in Fig. 3).

### 2.2 Post-operative

**Fig. 4:**
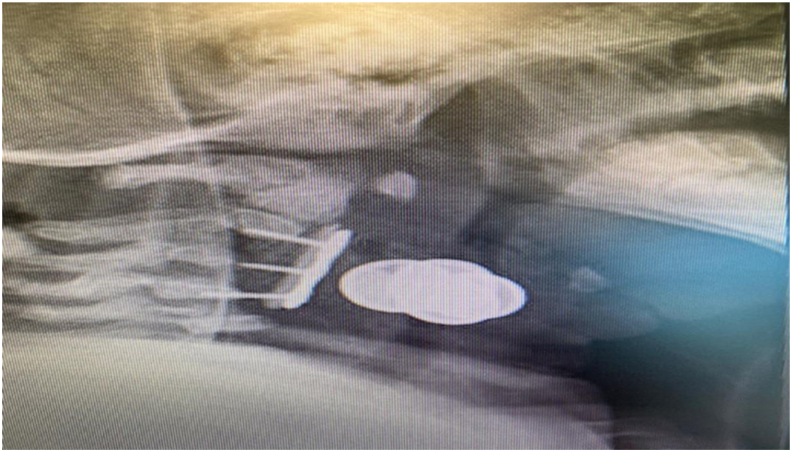
A cervical spine x-ray was done 4 months after follow-up showing properly placed plate and screws over the body of C2 iliac bone graft and C4.

The patient was placed on abduction brace (pavlik harness) and the brace was used for 3 months to prevent early collapse. A cervical spine x-ray was done 4 months after follow-up showing properly placed plate and screws over the body of C2 iliac bone graft and C4 (Shown in Fig. 4). The patient had noticeable improvement in neck spasticity and position.

## 3 Discussion

Larsen syndrome patients can present with multiple spinal abnormalities, but the most common and dangerous one is cervical kyphosis [7,8]. This can be very challenging to the spine surgeons as they have to take into consideration the age, individual presentation and severity of the disease [2]. We present a case of Larsen syndrome with cervical kyphosis. Our patient was the youngest patient reported in the literature to be operated on.

Madera et al. did a literature review of surgically treated cervical deformities in patients with Larsen syndrome from years the 1976 to 2008 [2]. Madera et al. reported a case of a 30-month-old boy who was asymptomatic. The patient underwent a combined anteroposterior decompression and fusion with external fixation. The treatment goals that the surgeons were aiming to reach were the probability of a successful fusion and to reduce the need for future surgery in addition to enhancing the cervical stability should the boy subsequently experience a traumatic fall [2]. Deora et al. did a review of all cases of surgically corrected cervical kyphosis in patients of Larsen syndrome. In their paper Deora et al. reported a case of a 15-year-old boy who had multiple deformities; one of them being a gross kyphotic deformity at C6 to C7 and atlantoaxial dislocation that disabled the patient from doing basic life activities. The patient underwent a transoral decompression f/b occipito-T1 fusion. His post-operative course was good as the patient was able to perform day to day activities [1].

The type of surgery is usually dictated by the severity and level of kyphosis but one thing we all agree on is the need for early surgical intervention to prevent further deterioration [4,9]. Our patient was the youngest patient with cervical kyphosis to be operated on; a C3 level corpectomy was done and the patient was put in a brace. A four month x-ray follow-up was done and the patient was doing well.

The most important step in the management of this syndrome is surgical intervention; and the procedure done in order to achieve spinal stabilization can promise a better long term outcome and spare the patient the need for future procedures [1,2]. Our 6 month old patient showed excellent recovery and clear improvement on follow-up. Therefore, patients with Larsen syndrome can have good prognosis if they receive the appropriate management.

## 4 Conclusion

Larsen syndrome comes with many defects; especially spinal defects. The most severe one which can eventually lead to paralysis. Therefore, surgical intervention is the most important step in the management of these patients. In this paper, we report the youngest case of Larsen syndrome operated on and we aim to emphasize on the importance of early intervention since it can spare the patients future surgeries and can promote better outcomes.

## Ethical approval

Written informed consent was obtained from the patient's parents/legal guardians for publication of this case report and any accompanying images. A copy of the written consent is available for review by the Editor-in-Chief of this journal on request.

## Funding

There are no sponsors involved in the study.

## Author contribution

1) Mohammed Armouti: acquisition and analysis of the data.
2) Hawazen Hirbawi: analysis of the data and writing of manuscript.
3) Mutaz Jadaan: data analysis and revising the manuscript.
4) Hasan Hashem: revision of manuscript and contribution to the design of the work.
5) Baha'eddin A.Muhsen: acquisition and analysis of the data and final approval of the version to be published.

## Guarantor

Baha'eddin A. Muhsen MD.

*Division of Neurosurgery, Department of Surgery, King Hussein Cancer Center, Amman, Jordan. Email: Bmuhsen08@gmail.com Tel: +962777990888.

## Consent

Alterations do not distort scientific meaning.

## Data availability statement

The data that support the findings of this study are available from the corresponding author upon reasonable request.

## Provenance and peer review

Not commissioned, externally peer-reviewed.

## Declaration of competing interest

None.
